# [Corrigendum] Angiotensin II type 2 receptor-interacting protein 3a suppresses proliferation, migration and invasion in tongue squamous cell carcinoma via the extracellular signal-regulated kinase-Snai2 pathway

**DOI:** 10.3892/ol.2026.15830

**Published:** 2026-08-24

**Authors:** Tingting Zhao, Qianting He, Zhonghua Liu, Xueqiang Ding, Xiaofeng Zhou, Anxun Wang

Oncol Lett 11: 340–344, 2016; DOI: 10.3892/ol.2015.3898

Following the publication of the above paper, an interested reader has drawn the Editor's attention to the fact that, regarding the cell invasion and migration assay data shown in Fig. 3B and C respectively on p. 343, the ‘ATIP3a siRNA’ data panel in Fig. 3B and ‘Control siRNA’ data panel in Fig. 3C were apparently matching, suggesting that the data in this figure had been assembled incorrectly. Upon consulting the authors in this regard, they have realized that the representative fluorescence microscopy images of UM2 cells treated with control siRNA (corresponding to the migration experiment in Fig. 3C) were incorrectly selected during the preparation of the final composite figure. Specifically, the representative Control siRNA-treated UM2 migration panel in Fig. 3C had been inadvertently duplicated from the ATIP3a siRNA-treated UM2 invasion panel in Fig. 3B.

The revised version of Fig. 3, now featuring the correct data for the ‘Control siRNA’ data panel in Fig. 3C, is shown on the next page. The authors wish to point out that this error was confined to the representative fluorescence micrographs, and did not affect the corresponding cell-count quantification, statistical analyses, figure legend, interpretation or conclusions of the study. All the authors agree with the publication of this corrigendum, and they are grateful to the Editor of *Oncology Letters* for allowing them the opportunity to publish this. The authors also apologize to the readership for any confusion caused.

## Figures and Tables

**Figure 3.**
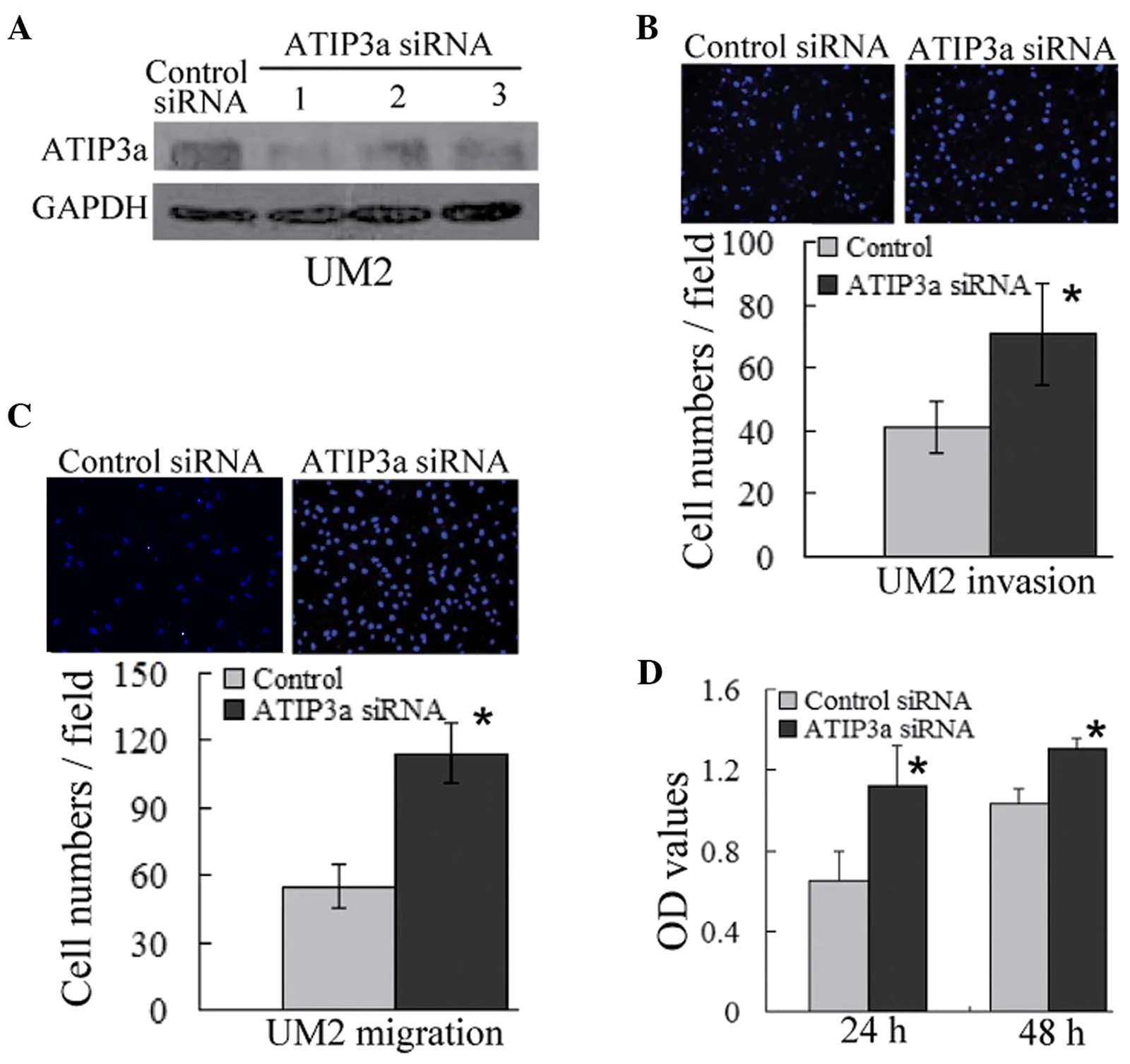
ATIP3a knockdown promoted the migration and invasion ability of tongue squamous cell carcinoma cells. (A) Significant reductions in ATIP3a protein levels were observed in the ATIP3a siRNA-transfected UM2 cells compared with the control siRNA-transfected cells. (B-D) ATIP3a knockdown promoted the (B) invasion, (C) migration and (D) proliferation ability of UM2 cells compared with that of the control siRNA-transfected UM2 cells. *P<0.05. ATIP3a, angiotensin II type 2 receptor-interacting protein 3a; siRNA, small interfering RNA; OD, optical density.

